# Unusual Mechanisms of Penile Amputation

**DOI:** 10.1155/2019/1582047

**Published:** 2019-12-26

**Authors:** Julian Fazi, David Adkins, Jennifer Knight, Adam Luchey

**Affiliations:** Department of Urology, West Virginia University, Morgantown, WV 26505, USA

## Abstract

Penile amputation is an uncommon and highly morbid injury. Many mechanisms have been reported ranging from self-mutilation and domestic violence to traumatic circumcisions. We present two unusual cases of traumatic penile amputation. An older gentleman endured extensive perineal trauma after being trapped underneath an industrial-sized lawnmower, and a young adolescent was bitten by an English bulldog and suffered amputation of the glans of his penis. These unique and very different cases of penile amputation highlight differences in operative managements, complications, and reconstructive possibilities.

## 1. Introduction

Penile amputation can present as an isolated injury or as part of an extensive perineal trauma requiring intensive care unit admission. Nevertheless this serious injury has major functional and psychological consequences [1]. Penile amputation injuries are rare, making it difficult to develop standardized management guidelines. The most common causes of penile amputations in adults are self-mutilation, domestic violence, and trauma. The most common causes in pediatrics are traumatic circumcision and automobile accidents [2, 3].

The original approach for penile replantation, which was first described in the late 1920's by Ehrich, was a macrosurgical technique that focused on aligning the major structures of the penis including the urethra, corpora spongiosa, corpora cavernosa, and glandular epithelium [4, 5]. This approach is dependent on the corporal sinusoidal blood flow [4]. The first microsurgical approach was completed in the late 1970's. This approach focuses on realigning smaller structures such as the dorsal neurovascular structures and the cavernosal arteries [1, 4]. The macrosurgical technique has higher rates of complications including skin necrosis, sensation deficits, erectile dysfunction, urethral strictures, and fistula formation when compared with the microsurgical approach [2–4]. Nonetheless, a variety of factors and situations help to determine if replantation is possible. These include degree of contamination, preservation of amputated segment, mechanism of injury, and ischemic time [2].

We report two unique cases of penile amputation. A 69-year-old man who rolled his industrial-sized lawnmower and experienced significant perineal trauma with near complete penile amputation after he became trapped underneath the lawnmower blade. The second case is a 12-year-old boy who had his glans bitten off by a dog. These rare and unusual mechanisms of penile amputation highlight differences in the management, complications, and reconstructive possibilities.

## 2. Case Presentations

### 2.1. Case One

**Figure 1:**
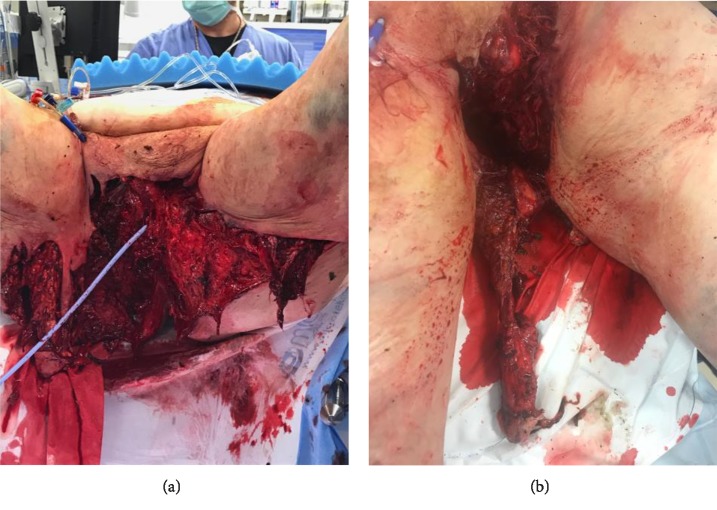
(a) The gaping perineal wound while lying in lithotomy position status post penectomy. A Foley catheter was inserted into the urethral stump. (b) Anterior view of perineal wound showing near complete amputation of the phallus.

The first case is a 69-year-old male who was found underneath an industrial-sized lawn mower in a rural area. The lawnmower had tipped as he was ascending a hill, and he was trapped underneath for an indeterminate amount of time. The patient suffered avulsion of his penis, scrotum, and testicles along with sustaining gaping perineal and left lower extremity wounds (Figure 1). He arrived in hemorrhagic shock. After resuscitation his immediate surgical management included hemostatic control, a descending loop colostomy, open J tube placement, and irrigation and debridement (I and D) of his perineal wound. His prostatic urethra was deemed intact and he had 4 cm of corpora cavernosa remaining bilaterally. A 16 French (Fr) Foley catheter was inserted into the urethra stump to empty the bladder and to prevent urine from further contaminating an already dirty surgical field. Bleeding was identified from the corporal stumps prompting ligation with 3-0 Vicryl horizontal mattress sutures. On the following day, his perineal and lower extremity wounds were irrigated and debrided. A 16 Fr suprapubic catheter was placed 2 centimeters proximal to the pubic symphysis allowing for urinary diversion from the large perineal wound. The remaining corporal stumps were found to be nonviable, prompting a total penectomy. The 4 cm corpora cavernosa stump was resected at the base of the penis, divided with LigaSure and suture ligated with 2-0 Vicryl. The corpora spongiosum and bulbomembranous urethra were ligated using interrupted vertical mattress with 0 Vicryl.

The patient returned to the operating room every other day for the next few weeks for I and D of his perineal wound. He underwent numerous nonurologic operations in the following months including open reduction and internal fixation of his left fibula, cephalomedullary nail insertion into his femur, and grafting of his perineal wound. Since the operations, the patient has returned for exchange of his suprapubic catheter, bladder spasms that are managed with anticholinergics, and urinary tract infections. The patient was most recently seen in the clinic two years following his initial injury. He is able to ambulate but continues to have intermittent bladder spasms controlled with oxybutynin. His suprapubic catheter is exchanged on a monthly basis.

### 2.2. Case Two

**Figure 2:**
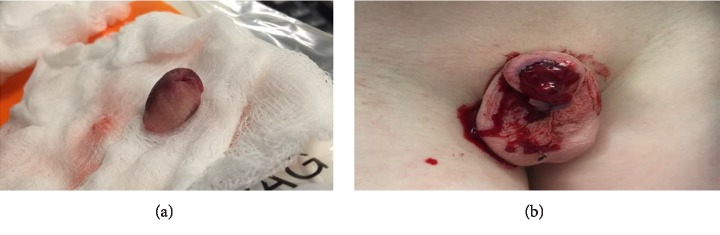
(a) The amputated glans. (b) The phallus proximal to the amputation.

**Figure 3:**
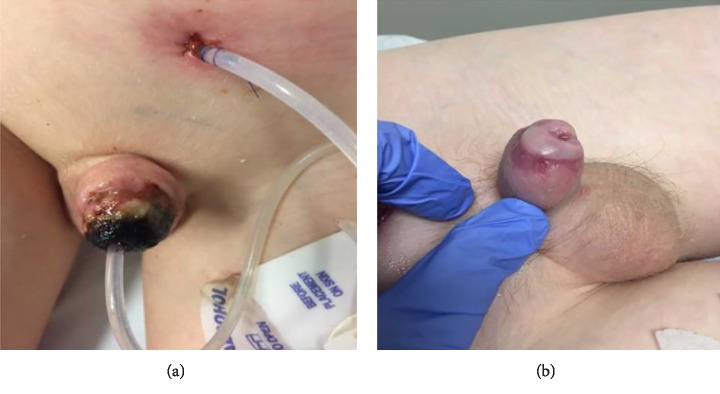
(a) Replantation of glans with suprapubic cystostomy tube. (b) Postoperative week ten with suprapubic and urethral catheters removed.

Patient two is a 12-year-old male who presented to the emergency department (ED) after his glans had been bitten off by an English bulldog (Figure 2). After hemorrhage control and gauze placement on the penile stump, the glans was wrapped in gauze and put in a bag of saline which was put in another bag of slush ice water. The patient was taken to the operating room, and it was determined that his corpora cavernosa were intact bilaterally. The operative team failed to identify any neurovascular structures conducive to microvascular repair. Hemorrhage control was attained using a vessel loop. Nonviable tissue was debrided from the distal shaft, and the distal shaft and glans were irrigated with copious amounts of normal saline and bacitracin. A 10 Fr Foley catheter was threaded through the penile meatus on the amputated glans and the urethra on the distal shaft to approximate, realign, and replant the glans. Immediate return of urine was noted when the catheter was advanced to the bladder. The urethral anastomosis was completed with 4-0 Vicryl sutures. The glans was reapproximated, with a macrosurgical approach, to the distal penile shaft with 4-0 Vicryl, and the glandular epithelium was reapproximated with 4-0 Monocryl sutures. The vessel loop was released. The glans was noted to be pink and purple without evidence of necrosis immediately following surgery. The patient was prescribed oral Augmentin for five days, Oxybutynin and Tamsulosin postoperatively. Mild superficial glans necrosis was noted two weeks postoperatively (Figure 3(a)) but the majority of the glans remained viable (Figure 3(b)). He was taken back to the operating room for placement of a 12 Fr suprapubic catheter and upsizing of his transurethral Foley catheter to 14 Fr to alleviate urinary retention and UTIs (Figure 3(a)). Cystoscopy noted a fibrotic urethra from the point of transection distally, without evidence of stricture. The Foley catheter was removed 6 weeks later. The patient performed clean intermittent catheterization (CIC) for one year postoperatively with the goal of preventing urethral stricture. CIC frequency was tapered down over the course of the year from every three-four hours initially to every eight hours, and he eventually was performing CIC on a weekly basis. Two years postoperatively the patient has a patent and nonstenotic urethra and meatus with a partially atrophic glans that is acceptable in size and cosmetic look. His urinary stream is normal and straight without any issues. The patient also reports that he is able to achieve erections and has sensation in the distal aspects of the penis.

## 3. Discussion

Penile amputation is a rare and devastating injury with a variety mechanisms reported. Some of the more common mechanisms include self-mutilation, domestic violence, trauma, and failed circumcision [2, 4, 6, 7]. Less common injuries include boating accidents, animal bites, agricultural machinery accidents among others [8–10]. A series of penile amputations in Thailand were maliciously performed in the 1970's by wives of unfaithful husbands [11]. Morrison et al. report that most amputations are complete amputations, and after replantation a majority of people have normal urinary function (97.4%), erections (77.5%), and sensation (68.4%) [2]. The most common complications after repair are skin necrosis (54.8%) and venous congestion (20.2%) [2].

Certain situations and factors make replantation more likely than others in the preoperative and operative settings. For example, success is very dependent on the length of ischemia that the amputated segment has endured; warm ischemia has a worse prognosis than cold ischemia [3]. Replantation has been reported after 16 hours of ischemia, and Jezior et al. suggest attempting replantation within 24 hours [1]. Other positive prognostic signs preoperatively are minimal contamination and preservation of the amputated segment [2]. Intraoperatively there is a greater chance of graft survival and preservation of erectile and urinary function with less postoperative complications if the microsurgical technique is used instead of the macrosurgical. Good venous outflow and return of normal color are good intraoperative and postoperative indicators of lasting tissue survival [2–4]. Furthermore, patients should be medically and psychologically stable before attempting replantation [1]. In our first case, reimplantation was not attempted given the severity of the trauma, lack of any clean margins, wound contamination, and the patient's hemodynamic instability.

In the setting of distal penile amputation involving only the glans, situations where vasculature cannot be identified or when instrumentation for microscopic surgery is not available, macroscopic replantation may be the only reconstructive option besides completion of a stump plasty [2, 12]. Amputation of only the glans is less common than more proximal amputations with one study citing glans amputations at 3.7% of total amputations [13]. Two adult cases of amputation at the glans, reported by Terayama et al., were repaired with stump plasty and experienced minimal postoperative complications. Normal urinary function, erectile function, and ability to have intercourse were ultimately achieved with one brief episode of urinary difficulty. One patient had reduced frequency of intercourse due to the shorter phallus causing an inferiority complex [13].

## 4. Conclusion

These cases contrast each other in mechanism, circumstances, and operative management. The 69-year-old male highlights a case where contamination, prolonged time of warm ischemia, severity of other injuries, and a poorly preserved phallus make replantation via a microsurgical or macrosurgical approach impossible. It also presents a unique mechanism of penile and perineal trauma not commonly cited. The 12-year-old male demonstrates a rare case of an amputation only involving the glans that was able to be macrosurgically replanted due to short time of cold ischemia and good tissue preservation.

## References

[1] Jezior J. R., Brady J. D., Schlossberg S. M. (2001). Management of penile amputation injuriess. *World Journal of Surgery*.

[2] Morrison S. D., Shakir A., Vyas K. S. (2017). Penile replantation: a retrospective analysis of outcomes and complications. *Journal of Reconstructive Microsurgery*.

[3] Babaei A. R., Safarinejad M. R. (2007). Penile replantation, science or myth? A systematic review. *Urology Journal*.

[4] Garg S., Date S. V., Gupta A., Baliarsing A. S. (2016). Successful microsurgical replantation of an amputated penis. *Indian Journal of Plastic Surgery*.

[5] Ehrich W. S. (1929). Two unusual penile injuries. *The Journal of Urology*.

[6] Fernando N., Facio J., Spessoto L. C. (2015). Penile replantation after five hours of warm ischemia. *Urology Case Reports*.

[7] Patial T., Sharma G., Raina P. (2019). Traumatic penile amputation: a case report. *BMC Urology*.

[8] Elmaraghi S., Chen T. J., Heckman J. E. (2019). Functional penile replantation after traumatic avulsion amputation below the pubis: a case report. *Microsurgery*.

[9] Dogra P. N., Gautam G., Ansari M. S. (2004). Penile amputation and emasculation: hazards of modern agricultural machinery. *International Urology and Nephrology*.

[10] Gomes C. M., Ribeiro-Filho L., Giron A. M., Mitre A. I., Figueira E. R., Arap S. (2001). Genital trauma due to animal bites. *Journal of Urology*.

[11] Bhanganada K., Chayavatana T., Pongnumkul C. (1983). Surgical management of an epidemic of penile amputations in Siam. *The American Journal of Surgery*.

[12] El harrech Y. E., Abaka N., Ghoundale O., Touiti D. (2013). Genital self-amputation or the Klingsor syndrome: successful non-microsurgical penile replantation. *Urology Annals*.

[13] Terayama T., Sakamoto T., Ikeuchi H., Tanaka Y. (2017). Self-penile glans amputation: a report of two cases. *Acute Medicine and Surgery*.

